# Serum levels of selenium and smoking habits at age 50 influence long term prostate cancer risk; a 34 year ULSAM follow-up

**DOI:** 10.1186/1471-2407-11-431

**Published:** 2011-10-07

**Authors:** Birgitta Grundmark, Björn Zethelius, Hans Garmo, Lars Holmberg

**Affiliations:** 1 Department of Surgical Sciences, Uppsala University, Uppsala, Sweden; 2 Department of Public Health and Caring Sciences/Geriatrics, Uppsala University, Sweden; 3 King's College London, Medical School, Division of Cancer Studies, London, UK; 4 Medical Products Agency, Uppsala, Sweden; 5 Regional Oncologic Centre of the Uppsala-Orebro region, Uppsala University Hospital, Uppsala, Sweden

## Abstract

**Background:**

Serum selenium level (s-Se) has been associated with prostate cancer (PrCa) risk. We investigated the relation between s-Se, smoking and non-screening detected PrCa and explored if polymorphisms in two DNA repair genes: *OGG1 *and *MnSOD*, influenced any effect of s-Se.

**Methods:**

ULSAM, a population based Swedish male cohort (n = 2322) investigated at age 50 for s-Se and s-Se influencing factors: serum cholesterol, erythrocyte sedimentation rate and smoking habits. At age 71 a subcohort, (n = 1005) was genotyped for *OGG1 *and *MnSOD *polymorphisms.

**Results:**

In a 34-year-follow-up, national registries identified 208 PrCa cases further confirmed in medical records. Participants with s-Se in the upper tertile had a non-significantly lower risk of PrCa. Smokers with s-Se in the two lower tertiles (≤80 μg/L) experienced a higher cumulative incidence of PrCa than smokers in the high selenium tertile (Hazard Ratio 2.39; 95% CI: 1.09-5.25). A high tertile selenium level in combination with non-wt rs125701 of the *OGG1 *gene in combination with smoking status or rs4880 related variation of *MnSOD *gene appeared to protect from PrCa.

**Conclusions:**

S-Se levels and smoking habits influence long-term risk of PrCa. Smoking as a risk factor for PrCa in men with low s-Se is relevant to explore further. Exploratory analyses of variations in *OGG1 *and *MnSOD *genes indicate that hypotheses about patterns of exposure to selenium and smoking combined with data on genetic variation in genes involved in DNA repair can be valuable to pursue.

## Background

Oxidative DNA base modification likely plays an important role in prostate carcinogenesis [1]. Both endogenous and exogenous antioxidants act interdependently in preventing clinically significant prostate cancer [2]. The selenoproteins active in oxidative DNA damage repair need the antioxidant selenium to be fully effective [3-8]. High serum selenium levels correlate with decreased incidence of cancer in general [9,10], and prostate cancer in particular [11-16], an association by some found to be mainly present in smokers [17]. Low serum selenium levels inversely relate to overall accumulated DNA damage [18] and selenoproteins are highly expressed in the prostate [19].

Despite that selenium thus theoretically may be a candidate anti-oxidant to use for prevention, the recent SELECT trial [20] - a large interventional study with selenium supplementation and/or vitamin E supplementation to reduce the risk of prostate cancer - was prematurely ended due to lack of effect. However, selenium may still be protective in a subset of men with specific genetic polymorphisms of the *MnSOD *gene [2] or in men with low baseline levels of serum selenium [21-23] which was not studied in SELECT but could be relevant to study further in light of recent findings[24].

The role of tobacco smoking in prostate cancer etiology is still unclear [25,26] but as smoking causes oxidative damage [27,28] and also is associated with lower serum selenium levels [29,30] a relation between smoking and prostate cancer seems biologically plausible.

Genes of interest to a relation between selenium and prostate cancer risk include those involved in DNA repair and oxidative stress. Oxidative stress triggers the base mutation 8-oxoguanine (OG) in DNA [31]. Single nucleotide polymorphisms (SNPs) in the gene coding for 8-oxoguanine glucosylase (*OGG1*), a selective base excision repair enzyme for OG-lesions [32], are associated with cancer progression [33,34] with evidence also for an association with prostate cancer [35-38]. Manganese superoxide dismutase (*MnSOD*) is the primary antioxidant enzyme in mitochondria. A functional SNP in the *MnSOD *gene, rs4880, modifies risk of prostate cancer associated with serum levels of pre-diagnostic selenium [2] although it not in itself was associated with prostate cancer risk [39]. *MnSOD *enzyme activity has a 56-fold range of values between polymorphic variants [40].

The aim of our study was to investigate the association between serum selenium levels at age 50 and later non-screening detected prostate cancer in a population based study with up to 34 years of individual follow up, and to assess if smoking status at baseline modified this association. In this cohort we also had information on factors with a potential to decrease serum selenium levels: Erythrocyte sedimentation rate (ESR), total serum cholesterol [41], and also of body mass index (BMI). In addition, we explored as a hypotheses generating analysis in a subcohort if polymorphism in the genes *OGG1 *and *MnSOD *influenced any effect of serum selenium.

## Methods

The study was approved by the Ethics Committee of the Faculty of Medicine at Uppsala University and written informed consent to participate was obtained from all participants.

The source population of the study is a cohort in the Uppsala Longitudinal Study of Adult Men (ULSAM). In 1970-1973, all male residents in Uppsala county in January 1970, born in 1920-24 (n = 2841) were invited to take part in a prospective health survey aimed at identifying risk factors for diabetes and cardiovascular disease. Eighty two per cent (n = 2322) of the invited men participated at baseline, at age 50, forming ULSAM [42].

**Figure 1:**
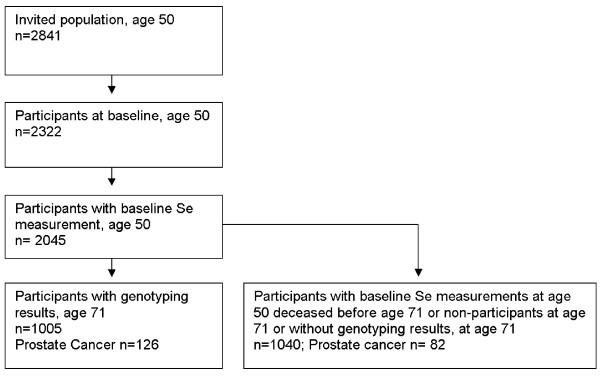
**Participant flow chart**. Flow chart of the investigated population of the study within the ULSAM cohort.

The serum selenium level at age 50 was determined for 2045 men in ULSAM and they constitute our study base (figure 1). After 21 years of follow-up, at age 71, the cohort was invited to a third cycle of investigation, performed from 1991 to 1995, including 1 221 of 1681 men still alive in January 1991. At this third cycle of investigation, blood samples for DNA extraction and later genotyping were collected from 1052 men giving consent for such sampling. Genotypes of *OGG1 *and *MnSOD *were available for1005 of the 1221 participants. The baseline mean serum levels of selenium, and two factors negatively correlated to selenium concentration: ESR and total serum cholesterol [41] did not differ between the 1005 genotyped and the full cohort of 2045 men who at baseline had their serum selenium levels determined. Therefore, the genotyped men were considered a representative sub-sample of the full cohort. Use of the drug clozapine may decrease serum selenium levels [43] but none of the participants reported use of this drug at age 50, i.e. concurrently with the blood sampling for selenium.

### Baseline examinations at age 50

Blood samples were drawn after an overnight fast. Serum concentrations were determined in samples that had been stored frozen (-70°C). Selenium was determined in serum using the graphite-furnace atomic absorption spectrometric method [44]. Fasting total cholesterol concentrations was performed on a Technicon Auto Analyzer type II [45] in 1981-82 on serum samples up until then stored in liquid nitrogen since 1970-73. ESR was determined by Westergren's method [46]. Based on personal interview reports the men were characterised as smokers or non-smokers (including ex-smokers) according to smoking status at age 50. Further sub-grouping of smoking data or stratifying by details of smoking history was not feasible due to sample size.

Weight, height and BMI (kg/m^2^) were measured under standardized conditions [47].

### Examinations at age 71

Four SNPs in proximity to the *OGG1 *gene were selected for genotyping; rs125701, based on the findings by [48] and another three SNPs distributed over the gene. The same method was used for the *MnSOD *gene where rs2758331 for technical reasons was used as proxy for rs4880, with a correlation coefficient (r^2^) = 0.92, [39,49,50] and further another five SNPs of the gene were investigated. The selected SNPs were genotyped at the SNP Technology Platform, Uppsala University, using the Golden Gate Assay (Illumina Inc.) [51]. The genotypes conformed to Hardy-Weinberg equilibrium according to a χ^2 ^test (p > 0.05). The reproducibility of the genotyping was 100% according to duplicate assays for 2.7% of the genotypes.

### Follow-up and outcome measures

Follow-up started at the examination in 1970-74 at age 50 and the censoring date for the present study was December 31, 2003, with a mean of 26.5 years of observation for the full cohort of 2045 men (54243 person years) and a mean of 31.1 years of observation for the genotyped 1005 men (31290 person years). To identify invasive prostate cancer (by International Classification of Diseases and Related Health Problems, 10th Revision, ICD-10; C61) we linked the unique personal identification numbers to the nationwide Population Register, the Cancer Register, the Hospital Discharge Register and the Causes of Death Register. The Cancer Register and the Causes of Death Register were established in 1958 while the Hospital Discharge Register covering all somatic inpatient health care was established in 1987. Reporting to these registries is compulsory. The coverage of prostate cancer in the Cancer Register is more than 95% [52] and for the other three registers 99% or more [53-55]. Identified prostate cancer cases were confirmed by reviewing the medical records whereby clinical details of the disease were collected (Table 1). In seven cases, where the medical records could not be retrieved, the diagnosis was verified by data in the National Prostate Cancer Registry, a nationwide clinical database started in 1997 [56].

**Table 1:** Clinical characteristics of the men diagnosed with prostate cancer (PrCa) in the full cohort and in the genotyped subcohort.

	PrCa in full cohort (n = 208)		PrCa in subcohort (n = 126)	
**Age at diagnosis: median (range)**	73	(57-83.0)	75	(59-82.0)

**Diagnosis confirmed by: n (%)**				

Histology	164	(78.8)	112	(88.9)
Cytology	33	(15.9)	11	(8.7)
Clinical examination only	3	(1.4)	1	(0.8)
Information missing	8	(3.8)	2	(1.6)

**Diagnosis suspected from: n(%)**				

PSA Screening	2	(1.0)	2	(1.6)
LUTS**	127	(61.1)	85	(67.5)
Other urological or skeletal symptoms	33	(15.9)	18	(14.3)
Incidentally discovered†	15	(7.2)	10	(7.9)
Other	7	(3.4)	6	(4.8)
Information missing	24	(11.5)	5	(4.0)

PSA-level***, ng/mL n (%)				

≤4	8	(3.8)	6	(4.8)
4.1-10	24	(11.5)	18	(14.3)
10.1-20	31	(14.9)	25	(19.8)
20.1-100	41	(19.7)	33	(26.2)
> 100	23	(11.1)	14	(11.1)
Information missing	81	(38.9)	30	(23.8)

**T-stage‡, n (%)**				

T0/T1ab	28	(13.5)	20	(15.9)
T1c	25	(12.0)	19	(15.1)
T2	89	(42.8)	55	(43.7)
T3-4	47	(22.6)	27	(21.4)
TX/Information Missing	19	(9.1)	5	(4.0)

**N-stage‡, n (%)**				

N0	32	(15.4)	22	(17.5)
N1	12	(5.8)	5	(4.0)
NX	142	(68.3)	90	(71.4)
Information missing	22	(10.6)	9	(7.1)

**M-stage‡in combination with PSA, n (%)**				

M0	88	(42.3)	61	(48.4)
MX, PSA ≤20	11	(5.3)	11	(8.7)
MX, 20 < PSA≤100	26	(12.5)	17	(13.5)
M1/PSA > 100	48	(23.1)	22	(17.5)
Information missing	35	(16.8)	15	(11.9)

Gleason score*, n(%)				

2-6	50	(41.3)	41	(45.6)
7	20	(16.5)	17	(18.9)
8-10	31	(25.6)	26	(28.9)
Information missing	20	(16.5)	6	(6.7)

^* ^WHO grade translated into Gleason score G1 = 2-6, G2 = 7, G3 = 8-10 in 42 cases

** Lower urinary tract symptoms.

*** Prostate specific antigen

†Incidentally discovered under investigation for symptoms not related to the urinary tract

‡As classified by the International Union Against Cancer (**UICC**)

The practice of PSA testing was during the period of follow-up restrictive both nationally and in the region of the Uppsala University Hospital. General screening is still not recommended and uncommon in men who, like in this cohort now, are well above 80 years of age.

### Statistical methods

The full cohort of 2045 men with full baseline measurements was analyzed for the main outcome of prostate cancer in relation to selenium levels and smoking. The 1005 genotyped men at age 71 were also analyzed separately to explore any effect modifications of polymorphisms in *OGG*-1 or *MnSOD*. Smoking status and BMI influence life span expectancy and constitute competing forces of mortality with prostate cancer. The men in the full cohort and in the genotyped subcohort were divided into tertiles according to their serum selenium concentrations, selenium influencing factors (Table 2) and BMI.

**Table 2:** Prostate cancer risk by subgroups

	Full cohort						Genotyped men					
	
	NonPrCa*		PrCa		PrCa-risk		NonPrCa		PrCa		PrCa-risk	
**Serum selenium(μg/l)**	**N**	**%**	**N**	**%**	RR**	95% CI***	**N**	**%**	**N**	**%**	**RR**	**95% CI**

≤70	675	(36.7)	84	(40.4)	Ref	-	295	(33.6)	48	(38.1)	Ref	-
70.1-81.0	588	(32.0)	65	(31.2)	0.89	(0.65 - 1.24)	299	(34.0)	46	(35.7)	0.93	(0.62 - 1.4)
81+	574	(31.2)	59	(28.4)	0.83	(0.60 - 1.16)	285	(32.4)	33	(26.2)	0.72	(0.46 - 1.12)

**Erythrocyte sedimentation rate, ESR(mm/h)**												

≤4.0	704	(38.3)	84	(40.4)	Ref	-	348	(39.6)	51	(40.5)	Ref	-
4.1-8.0	587	(32.0)	67	(32.2)	0.97	(0.70 - 1.33)	297	(33.8)	38	(30.2)	0.89	(0.58 - 1.35)
8+	545	(29.7)	57	(27.4)	0.88	(0.63 - 1.23)	234	(26.6)	37	(29.4)	1.07	(0.7 - 1.62)

**Total serum cholesterol(mmol/l)**												

≤6.24	560	(30.5)	76	(36.5)	Ref	-	288	(32.8)	48	(38.1)	Ref	-
6.25-7.27	624	(34.0)	64	(30.8)	0.77	(0.55 - 1.07)	295	(33.6)	40	(31.7)	0.82	(0.54 - 1.25)
7.27+	653	(35.5)	68	(32.7)	0.78	(0.56 - 1.08)	296	(33.7)	38	(30.2)	0.78	(0.51- 1.19)

**BMI(kg/m**^**2**^**)**												

≤23.4	605	(32.9)	61	(29.3)	Ref	-	296	(33.7)	39	(31.0)	Ref	-
23.5-26.0	567	(30.9)	78	(37.5)	1.34	(0.96 - 1.87)	285	(32.4)	50	(39.7)	1.31	(0.86 - 1.99)
26+	665	(36.2)	69	(33.2)	1.03	(0.73 - 1.46)	298	(33.9)	37	(29.4)	0.96	(0.61 - 1.5)

**Smoking**												

non-smoker	449	(24.4)	69	(33.2)	Ref	-	258	(29.4)	45	(35.7)	Ref	-
smoker	961	(52.3)	86	(41.3)	0.6	(0.44 - 0.83)	394	(44.8)	47	(37.3)	0.70	(0.46 - 1.05)
ex-smoker	427	(23.2)	53	(25.5)	0.82	(0.58 - 1.18)	227	(25.8)	34	(27.0)	0.87	(0.56 - 1.36)

Unadjusted relative risk (RR) of prostate cancer (PrCa) for baseline tertiles of selenium with 95% confidence intervals (CI), the two selenium level modifying factors ESR, total cholesterol, BMI and for smoking status in the full cohort and in the genotyped men. The lowest tertiles and the non-smokers were used as references.

* Prostate cancer

** Relative risk

*** Confidence interval

Risks of a) prostate cancer and b) death without a diagnosis of prostate cancer were calculated by means of cumulative incidence curves [57] censoring for end of follow up without events and considering the both types of endpoints a) and b) as competing events.

Competing risk proportional hazards models were determined for the sub-distribution of prostate cancer through competing risk regression [58] considering death without a diagnosis of prostate cancer as a competing risk. Hazard ratios derived from proportional hazards models were used as the estimator of relative risk (RR) of the effect of serum selenium divided into tertiles, smoking habits, and SNPs.

## Results

### Prostate cancer characteristics

During follow-up 208 men (10.2%) in the full cohort of 2045 and 126 of the 1005 genotyped men (12.5%) (*OGG1*), were diagnosed with prostate cancer.

Table 1 shows clinical characteristics for the men diagnosed with prostate cancer in the full cohort and in the genotyped subcohort. In the full cohort the mean age at diagnosis was 73 years. A large proportion of men in the full cohort, 78.8%, were diagnosed by biopsy, either by trans-rectal ultrasound (TRUS) guided core needle biopsies or by surgery, predominately after transurethral resection of the prostate. Seventy-six percent of the men in the full cohort experienced symptoms leading to medical consultation and a diagnosis of prostate cancer. Dominating symptoms leading to diagnosis were lower urinary tract symptoms, skeletal pain, impotence or deteriorating general condition. PSA measurement at diagnosis was available for 61.1% in the full cohort. In the full cohort eight men had a PSA value at diagnosis below 4 μg/L. Only 25.5% of the men had a non-palpable primary tumour (T0-T1c) at diagnosis while 22.6% had T3 and T4 tumours, 47.6% of men in the full cohort had no confirmed metastases or an unknown metastasis status with PSA below or equal to 20 ng/mL. Gleason scores - including WHO grades for cytology verified cases translated into Gleason score - was 6 or below in 41.3% of the men of the full cohort.

### Baseline measurements in relation to risk of prostate cancer

Table 2 presents the unadjusted relative risk (RR) of prostate cancer for baseline tertiles of serum selenium, ESR, serum total cholesterol, BMI and for smoking status in the full cohort and in the subcohort of genotyped men. The mean serum selenium level in the genotyped men was 77.4 μg/l, considered a normal level for Europe but low when compared to levels in North America. None of the point estimates for tertiles differed statistically significantly from unity. However, for the upper two tertiles of serum selenium, serum total cholesterol and ESR all estimates were numerically below unity with the exception of the highest tertile of ESR for the genotyped men. Trend tests for the estimates were all non-significant. The genotyped men in the upper tertile of serum selenium presented with the lowest risk estimate of developing prostate cancer (RR 0.72; 95% CI; 0.46-1.12). Adjustments by baseline tertiles of ESR and total cholesterol level did not influence the prostate cancer risk estimates for the tertiles of serum selenium (data not shown).

The non-smokers and men in the middle tertile of BMI (23.5-26.0) at 50 years of age, appeared to have a higher risk of developing prostate cancer in the full cohort as well as among the genotyped men (Table 2) although not to a statistically significant level for all of these groups.

### Smoking, Serum Selenium and Prostate Cancer

Since the results by smoking and BMI may be influenced by the increased risk of dying early among smokers and among men with BMI over 26.0 or BMI under 23.4, we estimated the cumulative incidence of non-prostate cancer death and prostate cancer occurrence by smoking status in the full cohort, stratified by serum selenium levels in the genotyped subcohort.

**Figure 2:**
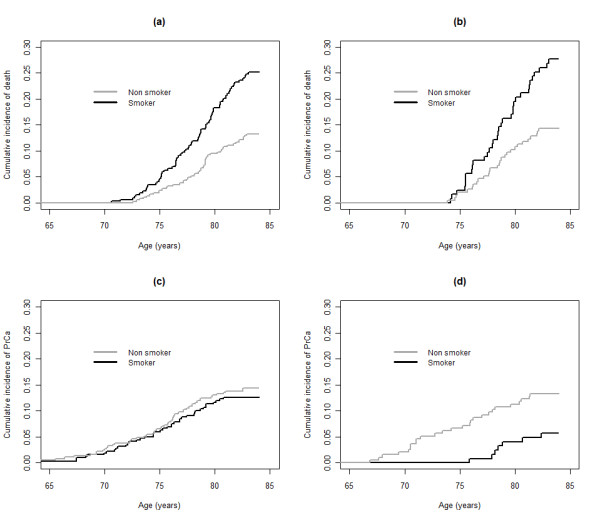
**a, b: Cumulative incidences of outcomes**. Cumulative incidences of death without prostate cancer for smokers (black line) and non smokers (grey line) by tertiles 1 and 2 combined (panel a) and tertile 3 (panel b) of serum selenium at baseline, 50 years of age in the genotyped subcohort. Figure 2 c, d: Cumulative incidences of prostate cancer for smokers (black line) and non-smokers (grey line) by tertiles 1 and 2 combined (panel c) and tertile 3 (panel d) of serum selenium at baseline, 50 years of age in the genotyped subcohort. Tertile serum selenium definitions; tertile 1 and 2 combined: ≤81 μg/L, and tertile 3: > 81 μg/L.

As illustrated in figure 2a and 2b, the cumulative incidence of death without prostate cancer in the genotyped subcohort was higher in smokers than in non smokers/ex-smokers combined, independently of tertiles of serum selenium (figure 2a low and middle serum selenium tertiles combined, figure 2b high serum selenium tertile). As would be expected from this result, due to their high incidence of early non-prostate cancer death, smokers should have a reduced cumulative incidence of prostate cancer, compared to non- and ex-smokers combined. This pattern is observed in Figure 2d for men in the upper tertile of serum selenium. In contrast to this however, for men in the combined middle and lower serum selenium tertiles shown in Figure 2c, the cumulative incidence of prostate cancer in smokers was equal to the cumulative incidence in non- and ex-smokers combined, which thus would indicate that smoking increases prostate cancer risk in men with lower serum selenium levels. These findings for the cumulative incidence are reflected in that smokers with serum selenium in the two lower tertiles (≤80 μg/L) experienced a RR of 2.39; (95% C.I. 1.09-5.25) for developing prostate cancer compared to smokers in the high serum selenium tertile.

The results of the competing risk analysis were similar to the results presented in figure 2, when the suggested critical serum selenium threshold level 100 μg/l was used instead of tertiles.

### The genotyped subcohort

**Figure 3:**
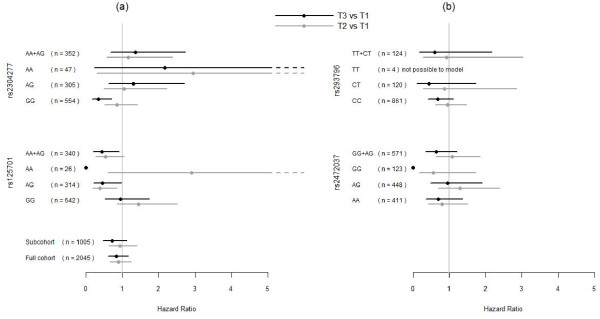
**a, b: Hazard ratios for prostate cancer by *OGG1 *genotype**. Hazard ratios (HR), with 95% confidence interval, for later diagnosis of prostate cancer in tertile 3 versus tertile 1(black line) and tertile 2 vs. tertile 1(gray line) of serum selenium levels at baseline, 50 years of age, by *OGG1 *genotypes. To the left (a) from top are results for SNPs rs2304277 and rs125701 and at the bottom for all genotyped men and the full cohort. To the right (b) from top are results for SNPs rs293796 and rs2472037. Tertile serum selenium definitions: tertile 1: ≤ 70 μg/L, tertile 2: 70.1- 81 μg/L and tertile 3: > 81 μg/L. "n" denotes number of participants in analysis.

We further explored if SNPs in the *OGG1 *or *MnSOD *gene modified the association between serum selenium levels, smoking status and prostate cancer risk presented in figure 2. The investigated SNPs in *OGG1 *per se did not influence the risk for prostate cancer on a statistically significant level. Taking serum selenium levels into account, presence of the A- allele of the SNP rs125701 in the *OGG1*gene, was observed to protect from prostate cancer (figure 3) in the high tertile of serum selenium compared to lower tertiles (p = 0.029). This was almost solely an effect in smokers with a HR of 5.8 (95% C.I. 2.13-16.1) with the caveat of this being a small subgroup of the whole cohort (n = 120).

**Figure 4:**
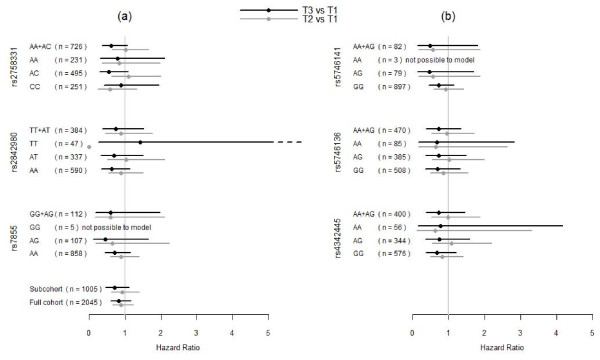
**a, b: Hazard ratios for prostate cancer by *MnSOD *genotype**. Hazard ratios (HR), with 95% confidence interval, for later diagnosis of prostate cancer in tertile 3 versus tertile 1(black line) and tertile 2 vs. tertile 1(gray line) of serum selenium levels at baseline, 50 years of age, by *MnSOD *genotypes. To the left (a) from top are shown results for SNPs rs2758331, rs2842980, rs7855. To the right (b) from top are shown results for SNPs, rs5746141, rs5746136 and rs4342445. Tertile serum selenium definitions: tertile 1: ≤ 70 μg/L, tertile 2: 70.1- 81 μg/L and tertile 3: > 81 μg/L. "n" denotes number of participants in analysis.

The investigated *MnSOD *SNP, rs2758331, used as a proxy for rs4880 similarly influenced the risk for prostate cancer to a near significant level when taking serum selenium levels into account (figure 4). Smoking did not influence the *MnSOD*-selenium level association.

## Discussion

In a study population where little PSA screening occurred, we found a previously not reported relation between serum selenium levels, smoking and prostate cancer risk: Smokers with serum selenium levels in the two lower tertiles (≤80 μg/L) experienced a higher cumulative incidence of prostate cancer than smokers in the high serum selenium tertile. In hypothesis generating explorative analyses, we found indications that the association between serum selenium levels and prostate cancer risk was modified by genetic polymorphisms, in the *OGG1 *gene (rs125701) and *MnSOD *gene (rs2758331- linked to rs4880).

The ULSAM cohort is population based and homogenous as regards the ethnic background and the age of the participants [42]. The participant rate was high and the follow-up up to 34 years almost complete through using linkage to national registers with high coverage. ULSAM has a thorough characterization of factors influencing serum selenium levels including information on smoking habits[42]. Only few prostate cancers were detected by PSA-screening and our results thus mainly pertain to clinically relevant prostate cancer with a high risk of progress.

For cancer studies, ULSAM is comparatively small and modest or weak associations may go undetected: our data indicate an overall modest inverse relation of serum selenium level with prostate cancer risk, which we could not substantiate. The serum selenium levels and co- variates were measured at a median of 23 years before diagnosis, a disadvantage, which however is shared with the other studies in the field [10,11,16]. In a Swedish population the range of selenium exposure is limited, with few high serum selenium level values. The size of the study implies that our analyses of the interaction of gene polymorphism and serum selenium levels are of an exploratory character. Another disadvantage is that a proxy SNP was used for the analysis of the main *MnSOD *polymorphism which made a detailed analysis on a molecular level unattainable.

Our finding of an increased risk for prostate cancer in smokers with low serum selenium is a new finding. As smoking increases oxidative damage, it is not biologically unreasonable. This relation may in other studies have been masked by the strong competing risk of concomitant early death from other causes, mainly cardiovascular disease, predominantly in smokers. If smoking is associated with both early death and risk of prostate cancer later in life, conventional time to event analyses may give a false impression that smoking protects from prostate cancer since methods traditionally employed assumes that censoring is non-informative. We noted and analysed a similar pattern for obesity in the ULSAM cohort [59]. This type of competing risk problem may be particularly evident in this study representing a pre-screening era where the median age at diagnosis of prostate cancer was 73 years of age. Thus, a screening program for prostate cancer introducing long lead times may alter this pattern.

Our hypothesis generating results that serum selenium levels and the polymorphic genes *OGG1 *and *MnSOD *involved in the protection from oxidative stress, act concurrently in the defence of prostate cancer development are in accord with previous knowledge [2,11,12,15,16,60]. A recent nested case-control study within the Physicians Health Study [24] analyzing polymorphisms within the selenoprotein gene, SEP15 found genetic variants associated with prostate cancer mortality and also modifying the association of serum selenium with prostate cancer survival. If, as the recent report suggest [24] and our study may be interpreted, only subgroups of men would benefit from high serum selenium levels, then beneficial effects of supplementation in interventional studies may pertain only to subgroups and therefore be difficult to detect. There was an overall lack of effect of selenium supplementation in the SELECT trial [20]. The trial inclusion criteria did neither enrich a study population with low baseline selenium levels nor patients defined by pre-specified genetic polymorphisms. The differences in results from the interventional study, ours and another recent observational study [24] are not contradictory but rather emphasize the possibility for interventional studies in predefined groups of men with low selenium concentrations and certain genetic profiles. Due to the limited size of our study, the impact of genetic variation in relation to smoking being a risk factor for prostate cancer was not possible to analyse further.

## Conclusions

Our results indicate that when competing risks of death are taken into account, middle age levels of serum selenium and smoking habits give long term information regarding the risk of prostate cancer presenting in a setting with a low prevalence of screening. We thus here identify a need for further development of statistical methods to take competing risk into account in prospective studies of risk factors for diseases with the prevalence peak late in life. The finding that smoking may be a risk factor for prostate cancer in men with low serum selenium levels is relevant to explore further. We found indications that patterns of exposure to selenium and smoking combined with data on genetic variation in genes involved in DNA repair for oxidative damage can be valuable to explore. Our findings are of relevance for the analysis of interventional studies with selenium supplementation.

## Competing interests

The authors declare that they have no competing interests.

## Authors' contributions

The authors jointly perceived and planned the study. BG collected the data for all men with prostate cancer. BG and HG carried out all analyses and HG supervised the statistical analyses including those of competing risk and of the genetic information. All authors jointly interpreted the results. BG wrote the first draft of the report and BZ and LH revised the report for intellectual content. BZ is guarantor for the ULSAM data and LH is the senior supervisor of the study. All authors approved the final manuscript.

## Pre-publication history

The pre-publication history for this paper can be accessed here:

http://www.biomedcentral.com/1471-2407/11/431/prepub
